# Intimate Partner Violence and Lower Relationship Quality Are Associated With Faster Biological Aging

**DOI:** 10.1037/pag0000581

**Published:** 2020-11-19

**Authors:** Kyle J. Bourassa, Avshalom Caspi, HonaLee Harrington, Renate Houts, Richie Poulton, Sandhya Ramrakha, Terrie E. Moffitt

**Affiliations:** 1 Center for the Study of Aging and Human Development, Duke University Medical Center, and Department of Psychology and Neuroscience, Duke University; 2 Department of Psychology and Neuroscience, Duke University, and Institute of Psychiatry, Psychology, and Neuroscience, King’s College London; 3 Department of Psychology and Neuroscience, Duke University; 4 Department of Psychology, University of Otago; 5 Department of Psychology and Neuroscience, Duke University, and Institute of Psychiatry, Psychology, and Neuroscience, King’s College London

**Keywords:** biological aging, romantic relationships, intimate partner violence, relationship quality

## Abstract

The characteristics of people’s relationships have relevance to health—high quality romantic relationships are associated with improved health whereas intimate partner violence is associated with poorer health. Recently, increased attention has been focused on the biological processes underpinning these associations. A geroscience approach—examining whether close relationship characteristics are associated with biological aging—would complement previous research focused on individual disease pathways. This study used participants from the Dunedin Study (*N* = 974) to investigate relationship characteristics and biological aging across almost 20 years, from age 26 to 45. Being involved in romantic relationships was associated with slower biological aging, β = −0.12, *p* < .001. This difference represented 2.9 years of aging over the two decades. Greater relationship quality was also associated with slower biological aging, β = −0.19, *p* < .001, whereas higher levels of partner violence were associated with faster biological aging, β = 0.25, *p* < .001. A 1 *SD* difference in these characteristics was associated with a difference of 1.0 and 1.3 years of aging over the two decades, respectively. Secondary analyses suggested that experiencing violence from a partner was more strongly associated with biological aging than perpetrating violence, and that the experience of physical violence was more strongly associated with aging than psychological violence. These findings suggest that the characteristics of romantic relationships have relevance for biological aging in midlife. Interventions designed to increase relationship quality and decrease partner violence could reduce future morbidity and early mortality by slowing people’s biological aging.

The presence of social relationships has consistently been linked to improved health outcomes and longevity when compared to the absence of close relationships (House, Landis, & Umberson, 1988). People who are more socially integrated are at lower risk of death and disease across the life span (Yang et al., 2016) and the magnitude of this association rivals other traditional risk factors for poorer health, such as a sedentary lifestyle and smoking (Holt-Lunstad, Smith, & Layton, 2010). It is not only the presence or absence of relationships that is health relevant. The characteristics of people’s social relationships, particularly their romantic relationships, are also linked to health outcomes. For example, greater relationship quality is linked to better health (Robles, Slatcher, Trombello, & McGinn, 2014), and greater exposure to intimate partner violence is associated with poorer health (Campbell, 2002; Coker et al., 2002). Although the presence and characteristics of romantic relationships are consistently associated with health, relatively less research has assessed whether these characteristics are associated with biological aging in early adulthood, before people generally develop chronic diseases and when such outcomes could potentially be prevented. The current study sought to address this gap by testing the association of romantic relationship characteristics and biological aging over a 20-year period spanning young adulthood through midlife.

The study of the biological processes underlying aging—termed *geroscience*—is a promising interdisciplinary area of research aiming to improve healthspan, the period of time in which people are free from disease and disability. Geroscience theory defines biological aging as having three features: (a) gradual physiological decline in one direction, (b) over years of time, (c) simultaneously involving the body’s multiple different organ systems. These features, in turn, lead to decreased functioning, an increase in age-related chronic diseases, and early mortality (Kaeberlein, 2013; López-Otín, Blasco, Partridge, Serrano, & Kroemer, 2013; Strong, Mathers, Leeder, & Beaglehole, 2005). Biological aging, though not a disease itself, represents a fundamental “common cause” of chronic diseases over the life span (Barzilai, Cuervo, & Austad, 2018; Kennedy et al., 2014; Moffitt, Belsky, Danese, Poulton, & Caspi, 2017). Slowing the rate at which biological aging occurs should reduce risk for many chronic diseases simultaneously, improving healthspan and reducing early mortality (Barzilai et al., 2018; Campisi et al., 2019; Justice et al., 2016). This promise led to the inclusion of geroscience in the 2020–2025 strategic research goals for the National Institute on Aging (2020), including a specific focus on “better understanding the effect of personal, interpersonal, and societal factors on aging.” Despite the potential that geroscience holds to better understand and improve human healthspan in the social and behavioral sciences, a majority of research in biological aging has focused on animal models. Application to humans has been slower (Belsky et al., 2017; Moffitt et al., 2017).

The study of close relationships could benefit from integrating a geroscience approach. A number of clinical health endpoints are associated with close relationships (Holt-Lunstad, 2018; Holt-Lunstad et al., 2010; Loving & Slatcher, 2013; Yang et al., 2016). However, research establishing specific biological mechanisms that explain these associations has been more elusive (Farrell & Stanton, 2019). Rather than focusing on specific chronic diseases or physiological pathways, biological aging represents a broad constellation of physiological, molecular, and cellular pathways that could help explain why people with fewer relationships or poorer quality relationships have increased morbidity and early mortality. The ultimate promise of this approach is that if relationship characteristics are associated with different rates of biological aging, interventions improving romantic relationship characteristics could provide a method to slow the development of chronic disease and disability.

Theories describing how romantic relationships affect health outcomes (Coan & Sbarra, 2015; Cohen & Wills, 1985; Karney & Bradbury, 1995; Sbarra & Hazan, 2008) can be broadly categorized into those focused on protective factors for people with high-quality social relationships and increased risk for people with poorer quality relationships. Slatcher and Selcuk (2017) organize several such theories within a *strength and strain* model, which includes both the positive and negative influences of romantic relationships and how they might interact to affect downstream health. This model presents many different pathophysiological pathways that might explain the association between relationships and health, including changes in endocrine, immune, and cardiovascular function (Slatcher & Selcuk, 2017). However, most empirical studies linking close relationships and health focus exclusively on clinical health endpoints or individual physiological pathways, such as immune function (Jaremka et al., 2013) or cardiovascular physiology (Bourassa, Ruiz, & Sbarra, 2019). Findings within specific physiological systems may obscure systemic changes in physiology observed across organ systems. For example, studies using allostatic load—a measure of health status that uses multiple biomarkers across physiological systems using clinical cutoffs—have found associations between relationship characteristics and physiological function across organ systems (Brooks et al., 2014; Rote, 2017). A geroscience approach would extend this work by incorporating continuously measured change in clinical biomarkers across multiple physiological systems, as described in the paragraph below. We would expect that biological aging would be slower among people who are in higher quality relationships (i.e., a strength), but faster among people in relationships with more partner violence (i.e., a strain), based on both the strength and strain model and previous empirical evidence linking relationship characteristics to physical health (Campbell, 2002; Robles et al., 2014).

The pace of aging (Belsky, Caspi, Houts, et al., 2015) is a recently validated method to quantify biological aging that fits well with a geroscience approach. The pace of aging models growth curves of physiological decline over four waves of biomarker data covering 20 years of functioning across seven different organ systems. This method is aligned with the geroscience definition of biological aging and allows for the direct assessment of the physiological processes associated with aging (Belsky et al., 2017), rather than inferring them from chronological age. Measuring the pace of aging over the period when most romantic relationships are formed and maintained could be used to test whether the presence and characteristics of romantic relationships are associated with biological aging, which could have implications for later morbidity and early mortality.

Studies of romantic relationships and health often assess relationship characteristics at a single time point, or select samples from a specific type of relationship status (e.g., continuously married). The strength and strain model implies that effects of romantic relationships on health should accumulate over time. Longitudinal investigations that include multiple measurements of relationship characteristics would better model the cumulative risk (Evans, Li, & Whipple, 2013) for poorer health that might be associated with relationship characteristics.

Previous studies are also limited in that they generally do not account for the types of early life experiences that might affect both romantic relationship characteristics and health. For example, adverse childhood experiences (ACEs) and childhood socioeconomic status (SES) are associated with both relationship quality and physical health in later life (Conger, Conger, & Martin, 2010; Voith, Anderson, & Cahill, 2020). It is possible that the association of social relationships and health could reflect social selection effects, rather than social causation—for example, people who are less healthy as children could face challenges in establishing high-quality relationships (Thompson, Marsland, Marshal, & Tersak, 2009)—and it is important to control for such alternative explanations as a result.

If romantic relationship characteristics are associated with the pace of aging across early adulthood and midlife, it would suggest that accelerated aging might be one pathway through which social relationships impact health outcomes. Romantic relationships are one well-defined and common example of a close relationship. Results from studying romantic relationships could be extended to social relationships more broadly. Romantic relationships are also unique in that there are empirically supported treatments to improve romantic relationship functioning (e.g., Christensen & Doss, 2017). Evidence that poorer quality relationships are associated with accelerated aging in young adulthood and midlife would suggest the opportunity for early intervention to prevent or slow the development of chronic disease and early mortality. This would align well with expectations that preventing accelerated aging earlier in life, rather than reversing aging in older adults, is a more promising avenue to improve healthspan and longevity (Barzilai et al., 2018).

## Present Study

The present study examined the associations between romantic relationship characteristics and biological aging in the Dunedin Study, a longitudinal cohort followed since birth. Study members reported on the quality of their relationship and their experience of partner violence at four occasions, ages 26, 32, 38, and 45. We tested the association between these relationship characteristics and study members’ biological aging—as assessed by the pace of aging—derived from biomarker panels assessed at the same four occasions. We predicted that being involved in romantic relationships (vs. being alone) and being involved in higher quality relationships would be associated with slower biological aging, whereas greater levels of partner violence would be associated with faster biological aging.

## Method

### Participants and Study Design

**Figure 1 fig1:**
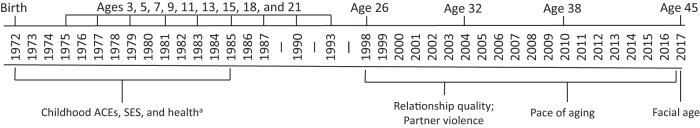
Outline of data collection time periods for the Dunedin Study. ACE = adverse childhood experience; SES = socioeconomic status. ^a^ Pack years and educational attainment were assessed to age 45.

Participants are members of the Dunedin Longitudinal Study, a longitudinal investigation of health and behavior in a representative birth cohort. The 1,037 participants (91% of eligible births) were all individuals born between April 1972 and March, 1973 in Dunedin, New Zealand, who were eligible on the basis of residence in the province and who participated in the first assessment at 3 years old (Poulton, Moffitt, & Silva, 2015). The cohort represents the full range of SES in the general population of New Zealand’s South Island and, as adults, matches the New Zealand National Health and Nutrition Survey on key adult health indicators (e.g., body mass index, smoking, and general practitioner visits) and citizens’ educational attainment of the same age from the New Zealand Census. The cohort is predominantly White (93%), matching South Island demographic characteristics (Poulton et al., 2015). Assessments were performed at birth; at ages 3, 5, 7, 9, 11, 13, 15, 18, 21, 26, 32, and 38 years; and, most recently (completed April 2019), at age 45 years, when 938 of the 997 participants (94.1%) still alive participated. For assessments, each participant came to the research unit for interviews and examinations. Written informed consent was obtained from cohort participants, and study protocols were approved by the institutional ethical review boards of the participating universities. The study’s preregistration materials detailing participant selection can be accessed online (Bourassa, 2019). The primary study sample included participants who reported whether they were in a relationship on at least two occasions during the age 26, 32, 38, or 45 assessments (*N* = 974, 49.3% women). Of the total sample, 883 (90.7%) reported on their relationship status during all four occasions, 62 reported on three occasions (6.4%), and 29 reported on two occasions (3.0%). The current study’s measurement space and design is provided in Figure 1.[Fig-anchor fig1]

### Measures

#### Biological aging

We assessed biological aging via two measures.

##### Pace of aging

Pace of aging was measured for each Dunedin participant with repeated assessments of a panel of 19 biomarkers taken at ages 26, 32, 38, and 45 years, as previously described (Belsky, Caspi, Houts, et al., 2015). The 19 biomarkers were: body mass index, waist–hip ratio, glycated hemoglobin, leptin, mean arterial pressure, cardiorespiratory fitness, forced expiratory volume in one second (FEV1), FEV1 to forced vital capacity ratio, total cholesterol, triglycerides, high-density lipoprotein cholesterol, apolipoprotein B100/A1 ratio, lipoprotein(a), creatinine clearance, urea nitrogen, C-reactive protein, white blood cell count, periodontal disease, and caries-affected tooth surfaces. Measures were taken in counterbalanced order across participants with the exception of blood, which was drawn at the same time of day for all participants at all four phases and dental examinations, which were conducted in the late afternoon at all four phases. Women who were pregnant at the time of a given assessment were excluded from that wave of data collection. Change over time in each biomarker was modeled with mixed-effects growth models, and these rates of change were combined into a single index scaled (within sex) in years of physiological change occurring per one chronological year (Belsky, Caspi, Houts, et al., 2015). Briefly, the biomarkers were standardized to a mean of 0 and *SD* of 1 based on the age 26 distributions. Mixed effect growth models were used to estimate each participants’ slope over the four study occasions for each biomarker individually. These slopes were then summed and scaled so that 1 year of chronological age equated roughly to 1 year of average change in physiological functioning in the sample. See the online Supplementary Materials 1 for additional details. Participants ranged in their pace of aging from 0.4 years of physiological change (slow aging) per chronological year to 2.4 years of physiological change per chronological year (fast aging).

##### Facial aging

Facial aging was included as a secondary measure of biological aging to complement the primary analyses using the pace of aging and provide additional support for the validity of study findings. Facial age is a valid biomarker of aging that predicts mortality above and beyond other relevant measures of health, as shown in cotwins discordant for facial age (Christensen et al., 2009). It is also an intuitive measure that indexes what other people perceive about a participants’ age. Facial age was based on two measurements of perceived age using ratings of each participant’s facial photograph by an independent panel of eight raters. First, age range was assessed by an independent panel of four raters, who were presented with standardized (nonsmiling) facial photographs of participants and were kept blind to their actual age. Raters used a Likert scale to categorize each participant into a 5-year age range (i.e., from *20–24 years old* up to *70+ years old*; interrater reliability = .77). Scores for each participant were averaged across all raters. Second, relative age was assessed by a different panel of four raters, who were told that all photos were of people aged 45 years old. Raters then used a 7-item Likert scale to assign a “relative age” to each participant (1 = *young looking*, 7 = *old looking*; interrater reliability = .79). The measure of perceived age at 45 years was derived by standardizing and averaging age range and relative age scores.

#### Romantic relationship characteristics

Study members reported on their romantic relationships during a relationship interview at ages 26, 32, 38, and 45.

##### Relationship covariates

Participants were asked about their relationship status and coded as either in a relationship (e.g., dating and involved in a relationship, cohabiting, or married) or not in a relationship (e.g., not currently dating or dating but not involved in a relationship) at each of the four phases, which allowed the construction of a count variable of the number of phases they reported being involved in a romantic relationship. In addition, we calculated the longest relationship length with a partner reported across the study occasions, which was 14.6 years (*SD* = 7.6, range = 0 to 30 years) on average.

##### Relationship quality

Relationship quality was assessed using nine questions from a previously validated interview at ages 26, 32, 38, and 45, which asked study members about shared activities and interests, balance of power, respect and fairness, emotional intimacy and trust, and open communication in their relationships (Robins, Caspi, & Moffitt, 2002). Answers to questions were coded 0 (*almost never*), 1 (*sometimes*), or 2 (*almost always*). Examples of items include, “We support each other during difficult times,” “We feel very close to each other,” and “I can count on my partner to help me.” These items were summed at ages 26, 32, 38, and 45, and then averaged to create an overall measure of relationship quality over the course of the study period. Study members’ reports of relationship quality correlated across the four phases, .18 < *r*s < .45, *p*s < .001. Possible scores ranged from 0 to 18, with higher scores representing greater relationship quality. Study members’ relationship quality was generally high across the four occasions, with a mean score of 15.9 out of 18, or 88.3% of the maximum score possible (*SD* = 2.2, range 6.3 to 18).

##### Partner violence

Intimate partner violence was assessed using the Dunedin Study Abuse Scales (see Ehrensaft, Moffitt, & Caspi, 2004; Magdol, Moffitt, Caspi, & Silva, 1998; Moffitt et al., 1997), a 33-item scale that used the items from the Conflict Tactics Scale–Revised (CTS-R; Straus, 1990), as well as additional items. The Physical Abuse scale included the nine physical violence items in the CTS-R, plus four additional items capturing other physically abusive behaviors (e.g., “Over the last year, did a partner ever push, grab, or shove you,” and “hit, or try to hit you with something.”). The Psychological Abuse scale consisted of two items from the CTS-R and 18 additional items capturing controlling, terrorizing, demeaning, and other psychologically abusive behaviors (e.g., “Over the last year, did a partner ever insult or shame you in front of others,” and “Humiliate (or ridicule) you.”). The full list of questions and instructions is included in the online Supplementary Materials 2. Study members were asked about their experience of partner violence victimization, as well as their perpetration of these 33 behaviors over the past 12 months in their romantic relationship. Study members reported whether each behavior occurred or not (0 = *not present*, 1 = *present*), and these 66 values were summed to create an overall score for partner violence at each age, with higher scores representing greater partner violence. These scores were summed across the four phases to create an overall index of intimate partner violence (*M* = 15.4, *SD* = 17.3, range = 0 to 131). Study members’ reports of partner violence correlated across the four phases, .33 < *r*s < .58, *p*s < .001. In addition to a total scale score, we also created subscales that assessed victimization versus perpetration, as well as subscale that assessed the experience of psychological violence versus the experience of physical violence. The correlations among these subscales are included in Table S1 in the online supplementary materials.

#### Childhood characteristics

We report three features of study members’ childhoods that were used as covariates to control for the types of early life, prerelationship experiences that might affect romantic relationship characteristics and health.

##### Adverse childhood experiences (ACEs)

As previously described (Reuben et al., 2016), archival study records from the first 15 years of study members’ lives were reviewed by four independent raters to determine whether study members experienced 10 ACEs identified in the Centers for Disease Control and Prevention (CDC) ACE study (CDC, 2016). This included five types of child harm (physical abuse, emotional abuse, physical neglect, emotional neglect, and sexual abuse) and five types of household dysfunction (incarceration of a family member, household substance abuse, household mental illness, loss of a parent, and household partner violence). As reported in more detail elsewhere (Reuben et al., 2016), interrater agreement across all ACEs between the four raters averaged a kappa of .79 (range = .76–.82). The mean count of ACEs in the sample was 1.1 events (*SD* = 1.2). Counts greater than four were recoded to four, in line with the CDC ACE study (CDC, 2016).

##### Childhood socioeconomic status (SES)

The SES of participants’ childhood families was measured using the 6-point Elley-Irving Socioeconomic Index for New Zealand (Elley & Irving, 1976). Childhood SES represented the average of the highest SES level of either parent across the assessments of cohort families from the study member’s birth through age 15 (*M* = 3.8, *SD* = 1.1, range = 1 to 6).

##### Childhood health

Study members’ childhood health was assessed using a panel of biomarkers and clinical ratings taken at phases spanning from birth to age 11. As described in more detail elsewhere (Belsky, Caspi, Israel, et al., 2015), children’s overall health was rated by two Dunedin Research Unit staff members at ages 3, 5, 7, 9, and 11 years based on review of birth records and assessment dossiers. These reports included a pediatric clinician’s assessments and reports of infections, diseases, injuries, hospitalizations, and other health problems collected from children’s mothers during standardized interviews. Clinical tests of motor development and measures of body mass, triceps and subscapular skinfold thickness, resting blood pressure, FEV1, and the ratio of FEV1 to forced vital capacity were also included. These assessments were averaged to create a standardized score (*M* = 0, *SD* = 1, range −2.5 to 2.5) of childhood health, with higher scores representing more health problems.

#### Educational attainment

Education was assessed using the highest level of education that study members reached by age 45. Scores were coded such that higher scores represented greater educational attainment: 0 = *no school certification* (14.7% of the sample), 1 = *school certification only* (14.4%), 2 = *high school equivalent* (40.1%), and 3 = *bachelor’s degree or higher* (30.7%).

#### Smoking history

Smoking behavior may be correlated with relationship characteristics and has direct health relevance, so smoking was assessed as an additional covariate. Study members were asked about their smoking history during study phases from age 15 to 45. The total amount of cigarettes smoked was used to compute an individual’s number of pack years at age 45 (mean at age 45 = 7.3, *SD* = 10.7). A pack year is equal to smoking one pack of cigarettes (20 cigarettes) per day for 1 year, or smoking two packs per day for half a year, and so forth (Thomas, 2014).

### Data Analysis

Study members reported on their romantic relationship status, quality, and partner violence at four phases of the Dunedin Study—ages 26, 32, 38, and 45. This yielded a 20-year relationship history for each study member, from early adulthood to midlife. Study members who reported on their relationships at least twice across the four phases (*n* = 974) were included in the first models examining the associations of relationship status and the pace of aging. We then tested associations of relationship quality and partner violence with the pace of aging among study members who reported on these relationship characteristics during at least two study phases (*n* = 909). We used independent multiple regression models that controlled for sex in all models. We then added relevant covariates in three separate steps: (a) relationship status covariates—phases with a relationship (2, 3, or 4) and longest length of relationship across the assessment periods; (b) childhood covariates—number of ACEs, childhood SES, and childhood health; and (c) additional covariates—educational attainment and smoking. Table 1 shows the associations of these covariates with the relationship characteristics and aging outcomes in the study. The covariates used in the current study were largely associated with study members’ relationship characteristics and biological aging.[Table-anchor tbl1]

After assessing our primary hypotheses, we carried out a series of secondary analyses. First, we tested the strength of association for different types of partner violence (the experience of violence victimization from an intimate partner as compared to the perpetration of violence; the experience of psychological violence as compared to physical violence from an intimate partner). Second, we tested the independent associations of relationship quality and partner violence with pace of aging when including both variables in the same model. Third, we examined the pace of aging among people who were in different kinds of relationships. Fourth, we reestimated the primary models using study members’ facial age—rather than pace of aging—as a secondary aging outcome. Finally, we tested latent growth curve models to explore whether change over time in relationship characteristics might also be associated with aging. To account for missing data in our models, we used full information maximum likelihood in MPlusVersion 8.3 (Muthén & Muthén, 2012). This method incorporates all available data and produces estimates that outperform other missing data treatments when data are missing at random (Graham, 2009). Analyses reported here were checked for reproducibility by an independent data analyst, who recreated the code by working from the article and applying it to a copy of the original data set.

## Results

### Romantic Relationships Across 20 Years

**Figure 2 fig2:**
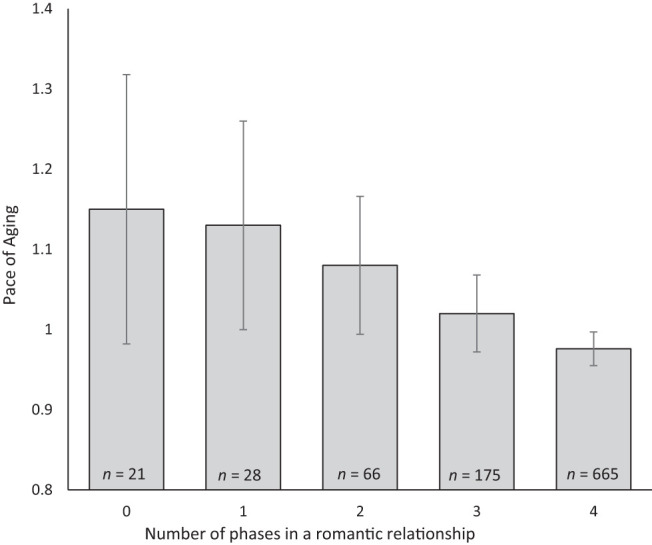
The mean pace of aging for study members organized by number of phases they were in a relationship. Nineteen people who did not have a valid pace of aging score were not included in this figure.

In total, 974 study members reported on their relationship status during at least two study phases. Most study members were in a relationship at all four phases (*n* = 665, 68.9%), 176 were in a relationship at three phases (18.1%), and 62 were in a relationship at two phases (6.4%). A small number of participants were never involved in a romantic relationship or involved in a relationship during one of the four study occasions (*n* = 55, 5.6%). These 55 participants had a significantly faster pace of aging, β = 0.12, 95% CI [0.05, 0.18], *p* < .001. The size of this effect represented a difference of 2.9 years of aging across the 19 years of the study, compared to those in relationship more often. The magnitude of this effect was similar for men and women—β for men = 0.11, 95% CI [0.01, 0.21], β for women = 0.12, 95% CI [0.02, 0.22]—and the interaction was nonsignificant, β = 0.05, 95% CI [−0.29, 0.40], *p* = .760. The association remained significant when accounting for childhood characteristics (childhood health, ACEs, childhood SES), β = 0.09, 95% CI [0.02, 0.15], *p* = .007, as well as educational attainment and smoking, β = 0.07, 95% CI [0.01, 0.1413], *p* = .015. Figure 2 illustrates the pace of aging for study members as a function of the number of study phases in which they were in a relationship.[Fig-anchor fig2]

### Relationship Quality and Aging

In the remaining analyses, we report on the study members who were in relationships and reported on their relationship characteristics during at least two study phases (*n* = 909). Table S2 in the online supplementary materials provides correlations among the study variables in this sample. Study members with higher average relationship quality had a slower pace of aging, β = −0.19, 95% CI [−0.25, −0.13], *p* < .001 (β = −0.18, 95% CI [−0.24, −0.12], *p* < .001, after controlling for 49 individuals [5.4% of the sample] diagnosed with cancer, heart attack, or diabetes). The size of this effect represented a difference of 1.0 years of biological age during the study for each *SD* change in relationship quality (2.2 points out of 18 possible). This association was of similar magnitude for men and women—β for men = −0.21, 95% CI [−0.30, −0.12], β for women = −0.17, 95% CI [−0.26, −0.08]—and the interaction was nonsignificant, β = −0.16, 95% CI [−0.66, 0.35], *p* = .542. The association between relationship quality and pace of aging remained significant when accounting for study members’ relationship characteristics (relationship length and number of phases in a relationship) β = −0.19, 95% CI [−0.25, −0.12], *p* < .001, childhood characteristics (childhood health, ACEs, and childhood SES) β = −0.16, 95% CI [−0.22, −0.09], *p* < .001, as well as educational attainment and smoking, β = −0.11, 95% CI [−0.18, −0.05], *p* < .001. Model results are presented in Table 2.[Table-anchor tbl2]

### Partner Violence and Aging

**Figure 3 fig3:**
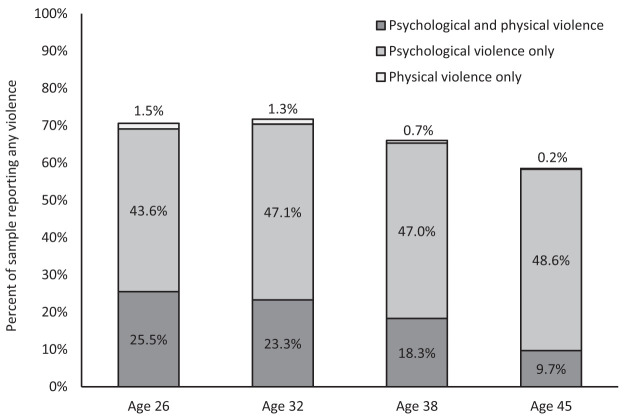
Percentage of the sample reporting intimate partner violence from age 26 to 45. The standard errors for these estimates are 1.55 at age 26, 1.54 at age 32, 1.61 at age 38, and 1.68 at age 45.

The prevalence of partner violence from ages 26 to 45 is visualized in Figure 3. Partner violence was relatively common—the rate of any violence (including both physical and psychological) within couples was highest at age 32 (71.7%). Prevalence decreased by age 45, at which 58.5% of the sample reported some type of violence. Psychological violence was more common than physical violence, and physical violence was rarely present in the absence of psychological violence (0.2 to 1.5% across phases). The rate of decline in physical violence from age 26 to age 45 was greater than the rate of decline in psychological violence, 25.5 to 9.7% (15.8% less) versus 69.1 to 58.3% (10.8% less).[Fig-anchor fig3]

Study members who reported higher mean levels of partner violence in their relationships had a faster pace of aging, β = 0.25, 95% CI [0.19, 0.31], *p* < .001 (β = 0.24, 95% CI [0.18, 0.30], *p* < .001, after controlling for 49 individuals [5.4% of the sample] diagnosed with cancer, heart attack, or diabetes). The size of this effect represented a difference of 1.3 years of biological age during the study for each *SD* change in partner violence (17.3 points). This association was of similar magnitude for men and women—β for men = 0.30, 95% CI [0.21, 0.38], β for women = 0.20, 95% CI [0.11, 0.29]—and the interaction was nonsignificant, β = 0.08, 95% CI [−0.13, 0.30], *p* = .455. The association between partner violence and biological aging remained significant when accounting for study members’ relationship characteristics, β = 0.25, 95% CI [0.19, 0.31], *p* < .001, childhood characteristics, β = 0.22, 95% CI [0.15, 0.28], *p* < .001, as well as educational attainment and smoking, β = 0.14, 95% CI [0.08, 0.21], *p* < .001. Model results are presented in Table 2.

### The Experience of Victimization Versus Perpetration of Partner Violence

Partner violence victimization and perpetration often co-occur in relationships. Dunedin Study members’ reports of experiencing victimization and perpetration were highly correlated, *r* = .68, *p* < .001, raising the question: does victimization or perpetration have more relevance to aging? Both victimization and perpetration were associated with faster pace of aging, β = 0.26, 95% CI [0.19, 0.32], *p* < .001 and β = 0.20, 95% CI [0.13, 0.26], *p* < .001, respectively. When including both the experience of victimization and perpetration of violence in a combined model, experiencing victimization was significantly associated with faster pace of aging, β = 0.23, 95% CI [0.14, 0.32], *p* < .001. This association remained significant after adjusting for study members’ relationship characteristics, childhood characteristics, educational attainment, and smoking, β = 0.16, 95% CI [0.07, 0.24], *p* < .001. Perpetration of violence, however, was not associated with study members’ pace of aging whether unadjusted, β = 0.03, 95% CI [−0.06, 0.12], *p* = .456, or adjusted for these covariates, β = −0.00, 95% CI [−0.09, 0.08], *p* = .957.

### The Experience of Psychological Versus Physical Violence Victimization

Given the relevance of partner violence victimization to accelerated aging, we next asked: what matters most, psychological or physical violence? Study members’ experiences of psychological and physical violence victimization by a partner were highly correlated, *r* = .72, *p* < .001. Both psychological and physical victimization were associated with faster pace of aging, β = 0.24, 95% CI [0.17, 0.30], *p* < .001 and β = 0.26, 95% CI [0.19, 0.32], *p* < .001, respectively. When including the experience of psychological and physical victimization in a combined model, the experience of more physical violence, β = 0.18, 95% CI [0.09, 0.27], *p* < .001, and the experience of more psychological violence victimization were both significantly associated with study members’ pace of aging, β = 0.11, 95% CI [0.02, 0.20], *p* = .018. The experience of both types of violence remained significantly associated with aging when adjusting models for study members’ relationship characteristics and childhood characteristics. However, when adjusting for educational attainment and smoking, the experience of physical violence was significantly associated with the pace of aging, β = 0.11, 95% CI [0.03, 0.20], *p* = .011, whereas the experience of psychological violence was not, β = 0.06, 95% CI [−0.02, 0.14], *p* = .142.

### Relationship Quality and Partner Violence in a Combined Model

Given that study members who reported higher quality relationships also tended to report lower levels of partner violence, *r* = −0.47, *p* < .001, the question remained whether relationship quality and partner violence were independently associated with biological aging. When combining relationship quality and partner violence in a single model, relationship quality and partner violence were independently associated with the pace of aging, β = −0.09, 95% CI [−0.16, −0.02], *p* = .013, β = 0.20, 95% CI [0.13, 0.28], *p* < .001, respectively. When also adjusting for study members’ relationship characteristics, childhood characteristics, educational attainment, and smoking, the effect for partner violence remained significant, β = 0.12, 95% CI [0.05, 0.19], *p* = .001, but the effect for relationship quality did not, β = −0.06, 95% CI [−0.13, 0.01], *p* = .099. An additional question was whether intimate partner violence might be more strongly associated with the pace of aging if relationship quality was also poor. To address this possibility, we tested whether relationship quality moderated the association of partner violence with biological aging. Relationship quality did not moderate this association, β = −0.04, 95% CI [−0.12, 0.04], *p* = .339.

**Figure 4 fig4:**
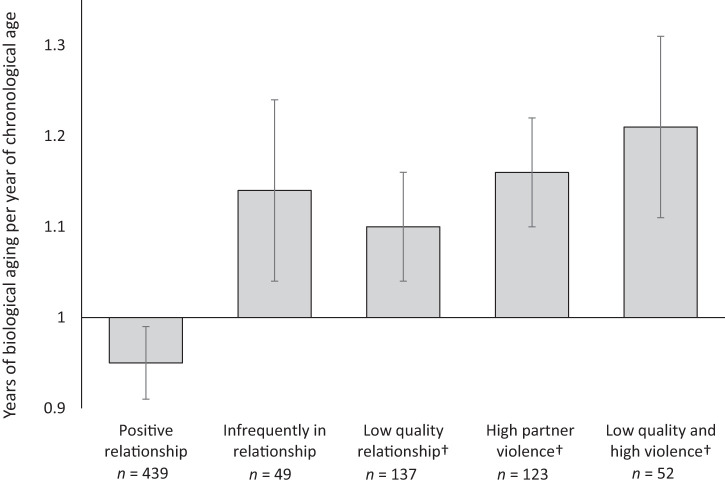
Comparing the mean pace of aging—indexed in years of biological aging per year of chronological age—for study members with different patterns of relationship status and characteristics. Groups included those who were in positive relationships (high quality and low violence), were infrequently in a relationship (less than 50% of study phases), were in lower quality relationships, were in relationships with more partner violence, and were in relationships with both lower quality and more partner violence. Independent group *t* tests revealed that people in positive relationships had a significantly slower pace of aging than those infrequently in a relationship (*d* = −0.61, *p* < .001), and those in low-quality and high-violence relationships (*d* = −0.82, *p* < .001), representing a difference of 3.6 and 4.9 years of biological aging over the course of the study, respectively. People infrequently in a relationship and people in low-quality and high-violence relationships did not significantly vary in their pace of aging (*d* = 0.19, *p* = .367). ^†^ These groups were not mutually exclusive.

A separate question was whether the pace of aging differed based on patterns of relationship characteristics. We calculated the mean pace of aging among groups based on the number of phases in which they were in relationships and the characteristics of those relationships (see Figure 4). People in higher quality relationships with low levels of partner violence had the slowest pace of aging (0.95, 95% CI [0.91, 0.97]) compared to people who were not in relationships (1.14, 95% CI [1.04, 1.24]), in lower quality relationships 1.10 [1.04, 1.16], in relationships with more partner violence, 1.16 [1.10, 1.22], or in relationships with both lower quality and more violence, 1.21 [1.11, 1.32] (as illustrated by the nonoverlapping 95% confidence intervals).[Fig-anchor fig4]

### Testing Associations With Facial Age at 45

Facial age was tested as a secondary outcome to investigate the associations of relationship characteristics with biological aging. Study members with better relationship quality had younger facial age, β = −0.10, 95% CI [−0.17, −0.03], *p* = .004. This effect remained when accounting for study members’ relationship characteristics and childhood characteristics, but became nonsignificant when educational attainment and smoking were included as covariates, β = −0.03, 95% CI [−0.10, 0.04], *p* = .389. Similarly, higher levels of partner violence were associated with older facial ages, β = 0.12, 95% CI [0.05, 0.18], *p* = .001. This effect remained when accounting for study members’ relationship characteristics and childhood characteristics, but became nonsignificant when educational attainment and smoking were included as covariates, β = 0.00, 95% CI [−0.06, 0.07], *p* = .927. Attenuation of the effect was primarily due to including smoking as a covariate. Full model results are presented in Table 3.[Table-anchor tbl3]

### Is Change in Relationship Quality and Partner Violence Over Time Associated With Aging?

In addition to average levels of relationship characteristics from age 26 to 45, these characteristics could have changed over time. Changes in relationship characteristics might be associated with the pace of aging, above and beyond people’s average levels. To investigate this possibility, we specified latent growth curves models for relationship quality and partner violence. In the first step, we included the relationship characteristic variables at each of the four occasions to determine the best-fitting growth curve. In the second step we correlated the resulting slope and intercept with the pace of aging. Models were assessed as having good fit if the RMSEA < .06, SRMR < .08, and CFI > .95 (Hu & Bentler, 1999).

For relationship quality, we began by fitting a linear model to the four occasions. The resulting model fit the data well, χ2 (5, *n* = 909) = 16.64, *p* = .005, SRMR = .05, CFI = .97, RMSEA = .051. We next correlated the pace of aging with the slope and intercept of relationship quality using this growth curve specification. The model including the pace of aging fit the data well for two of the three indices, χ2 (7, *n* = 909) = 27.46, *p* = .005, SRMR = .05, CFI = .94, RMSEA = .057. In this model, the pace of aging was associated with the intercept of relationship quality, β = −0.26, 95% CI [−0.36, −0.16], *p* < .001, but not the slope of relationship quality, β = −0.03, 95% CI [−0.16, 0.09], *p* = .581. In short, people’s relationship quality level was associated with their pace of aging, but change in people’s relationship quality over time was not.

We next examined partner violence, beginning by fitting a linear model to the four study occasions. The resulting model did not fit the data well for one of the fit indices, χ2 (5, *n* = 909) = 44.15, *p* < .001, SRMR = .05, CFI = .95, RMSEA = .093. Adding a quadratic growth factor improved model fit, but resulted in linear dependencies in the model. As a result, we specified a linear slope model that included quadratic growth values (0, 1, 4, 9). This improved model fit, though RMSEA remained above the cutoff for a well-fitting model, χ2 (5, *n* = 909) = 26.52, *p* < .001, SRMR = .04, CFI = .97, RMSEA = .069. We next correlated the pace of aging with the slope and intercept of partner violence using this growth curve specification. The model including the pace of aging fit the data well, χ2 (7, *n* = 909) = 27.01, *p* < .001, SRMR = .03, CFI = .97, RMSEA = .056. In this model, the pace of aging was associated with the intercept of relationship quality, β = 0.30, 95% CI [−0.36 −0.16], *p* < .001, but not the slope of relationship quality, β = −0.09, 95% CI [−0.16, 0.09], *p* = .055. In short, people’s mean level of partner violence was significantly associated with their pace of aging, but change in partner violence over time was not.

We next assessed whether moving the location of the intercept to one of the other three occasions (age 32, 38, or 45) altered these results. Changing the intercept did not alter any of the substantive findings in the model results for either relationship quality or partner violence. In each case, the pace of aging was significantly associated with the intercept of the relationship characteristics, but not the slope.

Finally, we assessed whether the strength of the associations of relationship quality and partner violence with the pace of aging differed depending on the age at which the relationship characteristics were assessed. The correlations between the relationship characteristics and the pace of aging were similar across occasions (Table S2 in the online supplementary materials), with the exception of relationship quality at age 45, which was not associated with the pace of aging.

## Discussion

The current study used a cohort of 974 adults to examine the associations between relationship characteristics and biological aging from young adulthood through midlife. The results revealed a number of notable findings. First, people who were in romantic relationships more often had slower biological aging compared to people who were less often involved in relationships. The difference between these groups represented a difference of 2.9 years of biological age over the course of the study. Second, people who were in higher quality relationships had relatively slower biological aging compared to those in lower quality relationships. Each 1 *SD* change in relationship quality represented a difference of 1.0 years of aging over the course of the study. Third, people with more partner violence in their relationships had faster biological aging compared to people with less partner violence in their relationships. Each 1 *SD* change in partner violence represented a difference of 1.3 years of aging over the course of the study. This association is particularly relevant given the common nature of partner violence (Ehrensaft et al., 2004; Magdol et al., 1998; Moffitt et al., 1997), which matches other epidemiological samples from countries such as the United States (Fagan & Browne, 1994; Magdol et al., 1997). Fourth, when examining different patterns and types of relationships, people who were in high-quality relationships with low levels of partner violence had the slowest aging, whereas people who were infrequently involved in relationships, who had lower quality relationships, or who experienced higher levels of partner violence all had similarly accelerated biological aging. Changes in relationship characteristics over time were not associated with biological aging, suggesting that it is the amount of cumulative exposure to these relationship characteristics over time, rather than whether relationship quality or partner violence increase or decrease, that has the most relevance to biological aging.

Fifth, relationship quality and partner violence were independently associated with the rate of biological aging, and these observed associations were robust to controlling for relationship covariates, childhood characteristics, educational attainment, and smoking. When including both relationship characteristics in the same model, however, controlling for smoking partially attenuated the associations with the pace of aging and fully attenuated the associations of relationship characteristics and facial age. The time course of smoking and relationship characteristics are unclear from the current study, but future studies would benefit from examining how health behaviors, such as smoking, may explain the association between relationships characteristics and biological aging. For example, it is possible that relationship stress led people to smoke more or have difficulty quitting smoking. This would be particularly relevant in the case of facial age, which could be more sensitive to differences in smoking status (Goodman et al., 2019). Finally, secondary analyses found that experiencing violence from a partner was more strongly associated with biological aging compared to perpetrating violence toward a partner. In addition, the experience of physical violence was more strongly associated with biological aging compared to the experience of psychological violence.

The current study had several notable strengths. First, assessing relationship characteristics and biological aging from age 26 to 45 provided a comprehensive assessment of relationships compared to studies that do not include repeated, longitudinal measurement. By using average levels of relationship quality and partner violence over a period of almost two decades, we were able to assess how the cumulative effects of these characteristics might be associated with aging, rather than assuming that an initial level of relationship quality or violence remained stable over time. Second, many studies of romantic relationships and health examine outcomes associated with relationship quality or partner violence independently. The current study assessed both relationship characteristics and included these variables in a single model to better characterize associations with biological aging.

Third, the design of the Dunedin Study allowed us to control for a variety of prerelationship characteristics (e.g., ACEs, childhood SES, childhood health) that might have contributed to the types of relationships people experienced and to their biological aging. Although statistical control cannot match experimental control, the ability to account for such potentially confounding variables is a strength in cases where such experimental control is not feasible or ethical (e.g., people cannot be randomly assigned to partners). Future studies could also test whether childhood characteristics might interact with adult romantic relationships to predict differences in biological aging.

Finally, using a biological aging approach to study the associations of relationship characteristics and health over young adulthood through midlife is a unique strength of this study. The majority of studies examining how social relationships might impact later health focus on a single physiological pathway or clinical health endpoint. In the case of social processes, it seems likely that there are a variety of ways relationship characteristics could “get under the skin.” Recent work linking poorer relationship characteristics to increased allostatic load across physiological systems supports this possibility (Brooks et al., 2014; Rote, 2017). In our study, biological aging was quantified using the pace of aging. By assessing gradual, coordinated physiological decline across seven different organ systems, this study examined change in biological processes associated with aging, rather than a specific health condition or chronological age. Our results suggest that measuring biological aging is a promising method to better understand how social relationships could be linked to physical health.

Our study findings are consistent with the strength and strain model (Slatcher & Selcuk, 2017) and extend this model by adding biological aging as a physiological mechanism that may explain how relationships could impact health outcomes. High relationship quality could act as a strength that buffers people against risk for accelerated aging, whereas partner violence could act as a strain, increasing risk for accelerated aging. In each case, differences in biological aging could translate to differences in later morbidity and early mortality. The results confirm the appeal and usefulness of applying a geroscience approach to study of close relationships.

How specifically might relationship quality and partner violence operate as strengths and strains? In the case of protective factors, many theories highlight the potential for high-quality relationships to modulate the stress–response system. The stress-buffering hypothesis (Cohen & Wills, 1985) suggests that high-quality social relationships are health-protective by buffering against the deleterious effects of stress. Similarly, coregulatory models of romantic relationships (Sbarra & Hazan, 2008) propose that having close others present allows individuals to better regulate their physiological reactions to stressful stimuli, both internal and external. Social baseline theory (Coan & Sbarra, 2015) highlights the value of close others in reducing the perception of threat in the environment, allowing for the conservation of biological resources in response to stress. Higher quality relationships could provide more of these protective benefits, slowing biological aging in turn.

In terms of strain, lower quality relationships have been theorized to affect health by increasing stress directly. The vulnerability–stress adaptation model (Karney & Bradbury, 1995) highlights that stress can interact with individual and couple-level vulnerabilities to negatively affect people. For example, romantic partners’ personalities can act as vulnerabilities that increase their stress and aggression toward their partner over time (Langer, Lawrence, & Barry, 2008; Moffitt, Robins, & Caspi, 2001; Robins et al., 2002). Higher levels of stress can directly result in poorer health (Richardson et al., 2012), and indirectly affect health by reducing relationship quality over time (Randall & Bodenmann, 2017; Robles et al., 2014). Such effects could be particularly strong in the case of extremely negative behaviors that can occur in romantic relationships, such as partner violence. The psychological and physical violence that occurs between couples has been shown to affect health, both in terms of the physical injuries, as well as indirect effects through increased stress and poorer relationship quality (Campbell, 2002; Campbell et al., 2002).

The results of the current study have clinical implications. These findings identify that people in lower quality relationships or relationships with high levels of partner violence (particularly experiencing physical violence) are at risk for poorer health due to accelerated biological aging. This presents a potential opportunity to intervene to improve relationship quality or reduce partner violence using empirically supported treatment, such as integrative behavioral couple therapy (Christensen & Doss, 2017) or acceptance and commitment therapy (Zarling, Lawrence, & Marchman, 2015). Improvement in relationship characteristics could slow the rate of biological aging, to the extent such effects are reversible. These efforts would deliver on the promise of geroscience by slowing the development of chronic diseases before such conditions develop and progress across a variety of physiological systems (Barzilai et al., 2018). Intervention studies would also help address whether the associations observed in this study might be reversible and could provide additional evidence as to specific mechanisms of action (Moffitt & The Klaus-Grawe 2012 Think Tank, 2013).

The study’s findings should be interpreted in light of its limitations. First, the study was correlational and did not allow us to directly test how relationship characteristics might have affected biological aging. It is possible these associations were due to physiological impacts on the stress response system, but alternative explanations are also plausible. For example, people who have accelerated biological aging may be more likely to select into lower quality relationships. It is equally possible that a third shared variable affects both relationship characteristics and biological aging. Such social selection is difficult to disentangle from social causation, though we made efforts to contend with such selection by controlling for prerelationship vulnerabilities (exposure to stress, lower SES, poorer health). Future studies using cotwin or sibling designs could help control for genetic predisposition to lifestyle or biological aging outcomes, as well as shared family background. Second, study members’ pace of aging and relationship characteristics were assessed over the same period of time, which did not provide temporal ordering. Methods of quantifying biological aging that provide point estimates would allow for temporal ordering over time, such as in cross-lagged panel models. Third, the current study draws on a birth cohort of individuals. It would be of great interest to study the association between relationship characteristics and biological aging within romantic couple dyads.

## Conclusions

The current study assessed the associations of relationship characteristics and biological aging in a cohort of 974 people. We found that people who were in romantic relationships tended to evidence slower biological aging. In addition, people who were in higher quality relationships had slower biological aging, and people with more partner violence in their relationships had faster biological aging, especially when experiencing physical violence from a partner. These findings support the value of using a geroscience approach to study social relationships, and suggest that accelerated biological aging might help explain the association between relationship characteristics and later health outcomes.

## Supplementary Material

10.1037/pag0000581.supp

## Figures and Tables

**Table 1 tbl1:** The Association of Study Covariates With Relationship Characteristics and Aging Outcomes

	Relationship characteristics				Aging outcomes			
*N* = 909	Higher relationship quality		Intimate partner violence		Pace of aging		Facial age	
Predictor variable	β	95% CI	β	95% CI	β	95% CI	β	95% CI
Relationship covariates								
Phases in a relationship	0.21**	[0.15, 0.28]	−0.01	[−0.08, 0.05]	−0.10**	[−0.16, −0.03]	−0.08*	[−0.14, −0.01]
Relationship length	0.32**	[0.26, 0.38]	−0.15**	[−0.21, −0.08]	−0.06	[−0.12, 0.01]	−0.02	[-0.09, 0.04]
Childhood covariates								
Adverse childhood events	−0.11**	[−0.18, −0.05]	0.17**	[0.10, 0.23]	0.18**	[0.12, 0.25]	0.11**	[0.05, 0.18]
Higher childhood SES	0.06	[−0.00, 0.13]	−0.12**	[−0.19, −0.06]	−0.22**	[−0.28, −0.16]	−0.23**	[−0.30, −0.17]
Poorer childhood health	−0.04	[−0.11, 0.02]	0.02	[−0.05, 0.09]	0.20**	[0.14, 0.27]	0.11**	[0.06, 0.18]
Additional covariates								
Educational attainment	0.18**	[0.12, 0.25]	−0.19**	[−0.26, −0.13]	−0.29**	[−0.35, −0.23]	−0.26**	[−0.32, −0.20]
Smoking	−0.19**	[−0.26, −0.13]	0.33**	[0.27, 0.39]	0.34**	[0.29, 0.40]	0.34**	[0.28, 0.40]
*Note*. This table reports bivariate correlations of the study covariates with relationship characteristics and aging outcomes. CI = confidence interval; SES = socioeconomic status.								
* *p* < .05. ** *p* < .01.								

**Table 2 tbl2:** The Associations of Relationship Characteristics With the Pace of Aging

*N* = 909	Model 1 Bivariate association		Model 2 Adding relationship covariates		Model 3 Adding childhood covariates		Model 4 Adding additional covariates	
Predictor variable	β	95% CI	β	95% CI	β	95% CI	β	95% CI
Relationship quality → Pace of aging	−0.19**	[−0.25, −0.13]	−0.19**	[−0.25, −0.12]	−0.16**	[−0.22, −0.09]	−0.11**	[−0.18, −0.05]
Intimate partner violence → Pace of aging	0.25**	[0.19, 0.31]	0.25**	[0.19, 0.31]	0.22**	[0.15, 0.28]	0.14**	[0.08, 0.21]
Perpetration versus victimization^†^								
Partner violence perpetration → Pace of aging	0.03	[−0.06, 0.12]	0.04	[−0.05, 0.13]	0.03	[−0.06, 0.11]	−0.00	[−0.09, 0.08]
Partner violence victimization → Pace of aging	0.23**	[0.14, 0.32]	0.23**	[0.14, 0.32]	0.21**	[0.12, 0.29]	0.16**	[0.07, 0.24]
Psychological versus physical victimization^†^								
Psychological violence victimization → Pace of aging	0.11*	[0.02, 0.20]	0.11*	[0.02, 0.20]	0.09*	[0.00, 0.18]	0.06	[−0.02, 0.15]
Physical violence victimization → Pace of aging	0.18**	[0.09, 0.27]	0.18**	[0.09, 0.27]	0.16**	[0.07, 0.25]	0.11*	[0.03, 0.20]
*Note*. Each model adds an additional set of covariates to the model results. Model 1 includes the predictor and sex, Model 2 adds phases in a relationship and relationship length, Model 3 adds adverse childhood experiences, childhood socioeconomic status, childhood health, and Model 4 adds educational attainment and pack years as covariates. CI = confidence interval.								
^†^ Results include both types of violence in the same multiple regression model. * *p* < .05. ** *p* < .01.								

**Table 3 tbl3:** The Associations of Relationship Characteristics With Facial Age

*N* = 909	Model 1 Bivariate association		Model 2 Adding relationship covariates		Model 3 Adding childhood covariates		Model 4 Adding additional covariates	
Predictor variable	β	95% CI	β	95% CI	β	95% CI	β	95% CI
Relationship quality → Pace of aging	−0.10**	[−0.17, −0.03]	−0.10*	[−0.17, −0.03]	−0.07*	[−0.14, −0.01]	−0.03	[−0.10, 0.04]
Intimate partner violence → Pace of aging	0.12**	[0.05, 0.18]	0.12**	[0.05, 0.19]	0.09*	[0.02, 0.15]	0.00	[−0.06, 0.07]
*Note*. Each model adds an additional set of covariates to the model results. Model 1 includes the predictor and sex, Model 2 adds phases in a relationship and relationship length, Model 3 adds adverse childhood experiences, childhood socioeconomic status, childhood health, and Model 4 adds educational attainment and pack years as covariates. CI = confidence interval.								
* *p* < .05. ** *p* < .01.
