# Supplementary material for: Intimate Partner Violence and Lower Relationship Quality Are Associated With Faster Biological Aging
Source: Psychol Aging. 2020 Nov 19;35(8):1127–39. doi: 10.1037/pag0000581 (PMC7712579; doi:10.1037/pag0000581)
Supplement: Supplementary file 1 [file pag0000581_supplemental.docx]

**Bourassa et al., Intimate Partner Violence and Lower Relationship Quality Are**

**Associated with Faster Biological Aging**

**Supplemental Information**

**Supplementary Materials 1.** Quantifying the Pace of Aging...…….……………………………….………….….……….2

**Supplementary Materials 2.** Dunedin Study Abuse Scales Items………………………………….………………………4

**Supplementary Table S1.** Correlations Among the Partner Violence Subscales………………………………………8

**Supplementary Table S2.** Correlations Among Primary Study Variables………………………………………….….….9

**Supplementary Table S3.** Relationship Characteristics and the Pace of Aging Across Study Occasions…10

**Supplementary Materials 1.** Quantifying the Pace of Aging

To measure biological aging, we evaluated correlated age-related decline in multiple physiological systems using the Pace of Aging. Over a 20-year period until midlife—at ages 26, 32, 38, and 45--we collected 19 biomarkers to assess changes in the function of cardiovascular, metabolic, renal, hepatic, immune, periodontal, and pulmonary systems, and quantified age-related decline shared among these systems.

We chose biomarkers of different physiological systems that are used in standard clinical practice (lipids), are gold standard (e.g., forced vital capacity in respiratory medicine), and that are reliably measured. All biomarkers were preregistered. We calculated each study member’s Pace of Aging in three steps. In the first step, we transformed each biomarker’s values to a standardized scale. For each biomarker at each wave, we standardized values according to the age-26 distribution. In the second step, we calculated each study member’s personal slope for each of the 19 biomarkers—the average year-on-year change observed over the 2-decade period. Slopes were estimated using a mixed effects growth model that regressed the biomarker level on age. The models took the form $B_{it}=\gamma_{0}+\gamma_{1}{Age}_{it}+\mu_{0i}+\mu_{1i}{Age}_{it}+\epsilon_{it}$, where *B_it_* is a biomarker measured for individual ‘*i*’ at time ‘*t*’, γ_0_ and γ_1_ are the fixed intercept and slope estimated for the cohort, and *μ_0i_* and *μ_1i_* are the “random” intercepts and slopes estimated for each individual ‘*i*’. In the third step, we combined information from the 19 slopes of the biomarkers to calculate each study member’s personal “Pace of Aging.” Because we did not have any *a priori* basis for weighting differential contributions of the biomarkers to an overall Pace of Aging measure, we combined information using a unit-weighting scheme. All biomarkers were standardized to have mean = 0, *SD* = 1 based on their age-26 distributions, so slopes were denominated in comparable units. We calculated each study member’s Pace of Aging as the sum of age-dependent annual changes in biomarker Z-scores: ${Pace of Aging}_{i}=\sum_{B=1}^{19} \mu_{1iB}$, where μ_1iB_ is the slope of biomarker ‘*B*’ for individual ‘*i*’. Because the Dunedin birth cohort represents its population, its mean and distribution represent population norms. We used these norms to scale the Pace of Aging to reflect physiological change relative to the passage of time. We set the cohort mean Pace of Aging as a reference value equivalent to the physiological change expected during a single chronological year.

**Supplementary Materials 2.** Dunedin Study Abuse Scales Items

**NOTE: Study members were given the form to self-complete unless they were poor readers (about 15% of the cohort).  If they were poor readers, they were read the questions, and they circled their responses in private, and placed their response sheet in a deposit box.**

In this session, we are also interested in your relationship with a romantic partner. This person could be of the same sex or opposite sex. Everything we discuss is completely confidential and can never be revealed to anyone, including your partner. And, remember, you’re free to decline any questions as we go along.

Next I’d like to ask you about the quality of your relationship. In the past year, did YOU ever...

1. Damage a household item or some part of the home out of anger towards the partner?
2. Deliberately dispose of, or hide, and important item of the partner’s?
3. Get very upset if dinner, housework, or home repair work was not done when you thought it should be?
4. Purposely damage or destroy the partner’s clothes, car, pet, or other personal possessions?
5. Insult or shame the partner in front of others?
6. Lock the partner in or out of the house?
7. Tell the partner that he/she could not work or study?
8. Try to stop the partner from seeing or talking to friends or family?
9. Restrict the partner’s use of the care or telephone?
10. Make threats to leave the relationship?
11. Try to turn family, friends, or children against the partner?
12. Order the partner around?
13. Frighten the partner?
14. Give in to the partner but plan revenge?
15. Humiliate (or ridicule) the partner?

Yes No

Yes No

Yes No

Yes No

Yes No

Yes No

Yes No

Yes No

Yes No

Yes No

Yes No

Yes No

Yes No

Yes No

Yes No

1. Physically twist the partner’s arm?
2. Treat the partner like he/she was stupid?
3. Threaten to hit the partner or throw something at him/her in anger?
4. Tell the partner he/she was ugly, fat, or unattractive?
5. Push, grab, or shove the partner?
6. Slap the partner?
7. Physically force sex on the partner?
8. Become abusive after using drugs or alcohol?
9. Shake the partner?
10. Throw, smash, hit, or kick something in disagreement?
11. Throw or try to throw the partner bodily?
12. Throw an object that could hurt, at the partner?
13. Choke or strangle the partner?
14. Kick, bite, scratch, or hit the partner with a fist?
15. Hit or try to hit the partner with something?
16. Beat the partner up (multiple blows)?
17. Threaten the partner with a knife or gun?
18. Use a knife or gun on the partner?

Yes No

Yes No

Yes No

Yes No

Yes No

Yes No

Yes No

Yes No

Yes No

Yes No

Yes No

Yes No

Yes No

Yes No

Yes No

Yes No

Yes No

Yes No

In the past year, did YOUR PARTNER ever...

1. Damage a household item or some part of the home out of anger towards you?
2. Deliberately dispose of, or hide, and important item of the yours?
3. Get very upset if dinner, housework, or home repair work was not done when he/she thought it should be?
4. Purposely damage or destroy your clothes, car, pet, or other personal possessions?
5. Shame you in front of others?
6. Lock you in or out of the house?
7. Tell you that you could not work or study?
8. Try to stop you from seeing or talking to friends or family?
9. Restrict your use of the care or telephone?
10. Make threats to leave the relationship?
11. Try to turn family, friends, or children against you?
12. Order you around?
13. Frighten you?
14. Treat you like you were stupid?
15. Give in to you but plan revenge?
16. Humiliate (or ridicule) you?
17. Physically twist your arm?
18. Push, grab, or shove you?
19. Slap you?
20. Physically force sex on you?

Yes No

Yes No

Yes No

Yes No

Yes No

Yes No

Yes No

Yes No

Yes No

Yes No

Yes No

Yes No

Yes No

Yes No

Yes No

Yes No

Yes No

Yes No

Yes No

Yes No

1. Become abusive after using drugs or alcohol?
2. Threaten to hit you or throw something at you in anger?
3. Tell you that you were ugly, fat, or unattractive?
4. Shake you?
5. Throw, smash, hit, or kick something in disagreement?
6. Throw or try to throw you bodily?
7. Throw an object that could hurt, at you?
8. Choke or strangle you?
9. Kick, bite, scratch, or hit you with a fist?
10. Hit or try to hit you with something?
11. Beat you up (multiple blows)?
12. Threaten you with a knife or gun?
13. Use a knife or gun on you?

Yes No

Yes No

Yes No

Yes No

Yes No

Yes No

Yes No

Yes No

Yes No

Yes No

Yes No

Yes No

Yes No

**Supplementary Table S1.** Correlations Among the Partner Violence Subscales

| *N* = 909 | (1) | (2) | (3) | (4) | (5) |
| --- | --- | --- | --- | --- | --- |
| Partner violence (1) | 1.0 |  |  |  |  |
| Experiencing partner violence (2) | .95 | 1.0 |  |  |  |
| Perpetrating partner violence (3) | .88 | .68 | 1.0 |  |  |
| Experiencing psychological violence (4) | .93 | .98 | .67 | 1.0 |  |
| Experiencing physical violence (5) | .80 | .85 | .58 | .72 | 1.0 |
| Note: All correlation *p*s were less than .001. | | | | | |

**Supplementary Table S2.** Correlations Among Primary Study Variables

| *N* = 909 | (1) | (2) | (3) | (4) | (5) | (6) | (7) | (8) | (9) | (10) | (11) | (12) |
| --- | --- | --- | --- | --- | --- | --- | --- | --- | --- | --- | --- | --- |
| Relationship quality (1) | 1.0 |  |  |  |  |  |  |  |  |  |  |  |
| Partner violence (2) | -.47 | 1.0 |  |  |  |  |  |  |  |  |  |  |
| Pace of aging (3) | -.19 | .25 | 1.0 |  |  |  |  |  |  |  |  |  |
| Facial age (4) | -.10 | .11 | .32 | 1.0 |  |  |  |  |  |  |  |  |
| Sex (5) | -.01 | .04 | -.01 | .04 | 1.0 |  |  |  |  |  |  |  |
| Phases in a relationship (6) | .21 | -.01 | -.09 | -.07 | -.02 | 1.0 |  |  |  |  |  |  |
| Longest length of relationship (7) | .32 | -.15 | -.06 | -.02 | -.15 | .50 | 1.0 |  |  |  |  |  |
| Childhood ACEs (8) | -.11 | .17 | .18 | .12 | -.01 | -.09 | -.13 | 1.0 |  |  |  |  |
| Childhood SES (9) | .07 | -.12 | -.22 | -.23 | .00 | .05 | .01 | -.26 | 1.0 |  |  |  |
| Childhood health (10) | -.04 | .02 | .20 | .11 | .02 | -.02 | -.00 | .06 | -.12 | 1.0 |  |  |
| Educational attainment (11) | .18 | -.19 | -.29 | -.26 | -.13 | .04 | .10 | -.24 | .40 | -.10 | 1.0 |  |
| Smoking in pack years (12) | -.19 | .33 | .34 | .34 | .08 | -.12 | -.19 | .29 | -.21 | .06 | -.42 | 1.0 |
| Note: ACEs = adverse childhood experiences, SES = socioeconomic status | | | | | | | | | | | | |

**Supplementary Table S3.** *Relationship Characteristics and the Pace of Aging Across Study Occasions*

| *N* = 909 | Pace of Aging | |
| --- | --- | --- |
|  | *r* *p* | |
| Mean relationship quality | -0.19 | . 003 |
| Relationship quality at age 26 | -0.10 | .001 |
| Relationship quality at age 32 | -0.15 | < .001 |
| Relationship quality at age 38 | -0.19 | < .001 |
| Relationship quality at age 45 | -0.04 | .332 |
|  |  |  |
| Mean partner violence | 0.25 | < .001 |
| Partner violence at age 26 | 0.17 | < .001 |
| Partner violence at age 32 | 0.19 | < .001 |
| Partner violence at age 38 | 0.21 | < .001 |
| Partner violence at age 45 | 0.15 | < .001 |
| Note: Mean values are averages include all four occasions | | |
